# An integrative review of e-learning in the delivery of self-management support training for health professionals

**DOI:** 10.1186/s12909-017-1022-0

**Published:** 2017-10-10

**Authors:** Sharon Lawn, Xiaojuan Zhi, Andrea Morello

**Affiliations:** Flinders Human Behaviour & Health Research Unit, Department of Psychiatry, Flinders University, PO Box 2100, Adelaide, South Australia 5001 Australia

**Keywords:** Self-management, Self-management support, E-learning, Blended learning, Integrative review

## Abstract

**Background:**

E-learning involves delivery of education through Information and Communication Technology (ITC) using a wide variety of instructional designs, including synchronous and asynchronous formats. It can be as effective as face-to-face training for many aspects of health professional training. There are, however, particular practices and skills needed in providing patient self-management support, such as partnering with patients in goal-setting, which may challenge conventional practice norms. E-learning for the delivery of self-management support (SMS) continuing education to existing health professionals is a relatively new and growing area with limited studies identifying features associated with best acquisition of skills in self-management support.

**Methods:**

An integrative literature review examined what is known about e-learning for self-management support. This review included both qualitative and quantitative studies that focused on e-learning provided to existing health professionals for their continuing professional development. Papers were limited to those published in English between 2006 and 2016. Content analysis was used to organize and focus and describe the findings.

**Results:**

The search returned 1505 articles, with most subsequently excluded based on their title or abstract. Fifty-two full text articles were obtained and checked, with 42 excluded because they did not meet the full criteria. Ten peer-reviewed articles were included in this review. Seven main themes emerged from the content analysis: participants and professions; time; package content; guiding theoretical framework; outcome measures; learning features or formats; and learning barriers. These themes revealed substantial heterogeneity in instructional design and other elements of e-learning applied to SMS, indicating that there is still much to understand about how best to deliver e-learning for SMS skills development.

**Conclusions:**

Few e-learning approaches meet the need for high levels of interactivity, reflection, practice and application to practice for health professionals learning to deliver effective SMS. Findings suggest that the context of SMS for patients with chronic condition matters to how health professional training is delivered, to ensure partnership and person-centred care. Further creative approaches and their rigorous evaluation are needed to deliver completely online learning in this space. Blended learning that combines e-learning and face-to-face methods is suggested to support SMS skills development for health professionals.

## Background

Health professional training has received much attention in the literature. Ongoing training is an expected part of meeting current registration and continuing professional development requirements for health professionals to support quality care and system improvement efforts. This training takes many forms depending on the workforce, the setting, available resources, service and system requirements, and the changing needs of patients, amongst other factors. The current healthcare landscape is one in which more traditional face-to-face forms of professional development training for health professionals are rapidly being supplemented or replaced with e-learning options using web-based technologies. E-learning is now seen as an integral method of learning for many health professionals; however, more understanding is needed about which formats are likely to best meet the learning outcomes needed for particular contexts, including the provision of self-management support for patients with chronic conditions.

### Defining e-learning

E-learning involves the delivery of education through Information and Communication Technology (ITC) using a wide variety of instructional designs and formats, and includes synchronous and asynchronous delivery [1–3]. It is often used synonymously with terms such as ‘internet-based learning’, ‘online learning’, ‘computer-assisted learning’ and ‘web-based learning’. There is significant diversity in what constitutes e-learning; it can include multi-media, CD-ROMs, webinars, virtual patients, web-based tutorials, interactive online modules with embedded quizzes, and discussion boards. A meta-analysis by Cook et al. [1] stressed that central to the definition is the use of the Internet and the computer to deliver information and interact directly with the learner; to replace, in part or completely, the human instructor. Sinclair et al. [3] provides a more detailed discussion of the definition, also stressing the importance of distinguishing between synchronous and asynchronous e-learning in order to more rigorously compare and measure outcomes of different instructional designs and formats. Synchronous e-learning is often mediated by human interaction between the learning and instructor using ITC and/or between learners who use ITC to interact and learn from each other in real time. In contrast, asynchronous e-learning involved more self-directed learning; it can occur at any time and place determined by the learner, and does not rely on a human facilitator being present.

### E-learning versus face-to-face learning

Central to the growing interest in e-learning as an alternative to traditional face-to-face learning formats is understanding how these formats impact the learner; how the learning experience may be similar or different. For example, a discussion involving nurses [4] found no difference in social and motivational maintenance preferences or study strategies; however, information processing habits differed with online learners showing significant differences in reflective observation and abstract conceptualization, and face-to-face learners being more likely to learn by doing. A US study with 20 nurse practitioners/nurse specialists comparing e-learning and simulation [5] found that participants preferred simulation, likely because the emphasis was on the direct translation of knowledge to practice, rather than knowledge for its own sake [5] (p.7). A systematic review of 19 qualitative studies about healthcare professionals’ experience of e-learning [6] found the key themes for effective learning were: peer communication, flexibility, support (informal from peers and from instructors), knowledge validation (being tested on what they had learned to consolidate knowledge, incentive to study), course presentation and design features, and use of real world case studies and resources that engaged them visually. A meta-analysis by Cook et al. [1] comparing e-learning with no intervention and non-internet delivered learning found large position effects of e-learning on knowledge outcomes, skills, behaviours and patient outcomes compared with no intervention, but these effects were heterogeneous and small when e-learning was compared with more traditional non-internet delivered learning. To understand these outcomes better, Cook et al. [2] then conducted a systematic review and meta-analysis of studies using quantitative measures to compare different types of e-learning concluding that interactivity, practice sessions, repetition and feedback were key features of e-learning associated with improved learning outcomes. A more recent review of randomized controlled trials [3] investigating the impact of e-learning on health professionals’ behaviour found mixed outcomes depending on the skills being taught and the approach to learning used. Firm conclusions were hampered by heterogeneity in instructional design and quality across the reviewed studies.

Many advantages of asynchronous e-learning have been noted in the literature, including its flexibility and the capacity for learning to be self-paced and traceable, catering to different learning styles, and enabling the learner to review as they need to, as well as e-learning’s capacity to overcome resource issues such as time and travel costs, and classroom-learner-staff availability issues [4, 7–12]. Mahmud et al., for example, argued that e-learning is more engaging than face-to-face and learners are more satisfied with it because it is more interactive and also because of design, navigation and ease of access [13]. A US evaluation of an e-learning case simulation library for nurses in aged care found that it was useful because it enabled learners to apply the cases to diverse clinical contexts [14]. Additionally a Japanese study comparing e-learning and face-to-face learning with 93 nurses [15] found that both groups demonstrated the same learning outcomes, but that the web group had three distinct differences. There was a lower dropout rate and greater flexibility, the online learning was attractive and affordable to a wider range of nurses, and was especially suited to independent and self-directed learners including those who were stronger in writing skills than in classroom discussion. Additionally, a systematic review of e-learning for nurses noted that the anonymity that it afforded to learners gave some greater confidence to reflect on and mediate their contributions to online discussions [16]. E-learning has also been promoted to meet the educational needs of rurally-based health professionals and their patients, as the tyranny of distance can isolate health professionals and make the option of face-to-face education difficult and often non-viable [17].

There are also many disadvantages of e-learning noted in the literature, including the need for increased responsibility and self-discipline to sustain motivation, concerns that those with poor study habits might fall behind, experience learner isolation and lack peer interaction to support learning, lack immediate support from teachers when questions/problems arise within an asynchronous context, and that standardized content could limit the ability for adaptations. It is worth noting that technology is intimidating for those with more limited technical skills, unreliable internet/technical access or platform instability potentially disrupting learning. Additionally, there may be distractions for the learner in the home environment where much e-learning takes place, and there may be significant upfront costs for those developing e-learning content [7, 9–12, 14, 18–20].

However, each of these reported advantages and disadvantages of e-learning must be understood in the context of significant heterogeneity of instructional designs and formats. This calls into question the extent to which firm conclusions can be drawn about the effectiveness of e-learning compared to more traditional approaches or blended learning as noted previously [1, 3].

Several studies have suggested that the context for e-learning matters. Shaikh et al., for example, have argued that e-learning, “needs to be used for the right task…not as a panacea for all medical education” [11] (p.710). E-learning might be useful for helping learners ascertain their level of knowledge and to reinforce and update existing learning; whereas, face-to-face learning might provide greater opportunities for discussion, debate and practicing skills. Hence, how and where content is delivered is important, and balancing the two is recommended [16]. Context might be important in other ways. For example, an Australian study examining 68 metro and rural nurse practitioners’ preferences for e-learning education [21] found that metro nurses had better access to high-speed internet than rural nurses (see also [22]). A UK study that surveyed 100 doctors about choice and approach to learning [23] found younger doctors who were high users of technology and more recently graduated appeared to have a deeper approach to learning with web-based approaches (see also [24]); however, they found no differences between gender, or metro/rural location. The wide diversity of how e-learning is delivered appears to be at the heart of problems with determining which types of instructional design and formats suit which contexts [2, 3].

Many studies have also been limited by the quality of their evaluation of the impact of e-learning; few studies have measured the impact of professional development on quality of care [25, 26]. A recent systematic review found no studies of sufficient quality that reported on effectiveness of e-learning on patient outcomes [3]. Many studies have focused on measuring learners’ satisfaction [27, 28]), perceived knowledge acquisition [29], perceived intention to change their practice [30], or self-reported practice change [22, 31, 32]. In summary, however, perceived intention or knowledge acquisition are at the lower end of evaluation and e-learning, if it is to maintain its relevance, should move towards more objective and rigorous evaluation methods [2, 3].

### E-learning and health professionals’ provision of chronic condition self-management support to patients

As healthcare delivery relies heavily on the quality of engagement and interactions with patients, particular arguments are warranted for how person-centred health professional training should be delivered. In particular, there is a lack of research on solely web-based technologies to train health professionals in chronic condition self-management support [33]. This paper aims to investigate the available evidence for the effectiveness of e-learning applied to the acquisition of the necessary knowledge, skills and attitudes of health professionals to effectively support self-management by patients with chronic and complex conditions. We begin by defining key terms and then provide a review of the literature on e-learning, generally, for health professionals.

Self-management is defined as what patients with chronic conditions do by taking action to cope with the impacts of their conditions [34, 35] There is growing evidence that patient self-management is effective at improving their emotional wellbeing, self-efficacy and changes to health behaviours [36]. However, patients’ competencies and confidence in self-managing health problems may be limited and may benefit from self-management support (SMS) provided by others (eg. services, health professionals, family, friends, carers) [34]. Historically, SMS was initially defined as educational and supportive interventions provided by health professions to systematically help chronically ill patients best meet their physical, social and emotional needs in long-term self-care management [37]. It includes provision of education and information to improve health literacy, practical support and also support with problem-solving, motivation and goal-setting and goal achievement. More recently, this definition has been broadened to recognize the more holistic support that is often also present from other service providers and service types, and from family and friends [34]. In the United States, patients have stated their desire for SMS, with this identified as a priority area for quality improvement, and added to national healthcare quality indicators [33, 37]. Effective SMS requires a balanced multi-modal approach including patient education and collaborative decision-making in order to achieve mutually acceptable self-management goals [38]. A review of more than 550 studies [39] showed that SMS by health professionals improved patients’ quality of life, symptoms and use of healthcare services (see also [40]).

To provide SMS, health professionals require effective training and education to facilitate their knowledge of supportive resources, collaborative skills to work within a multidisciplinary team, communication skills, better understanding of patients’ strengths and challenges, and insight into how living with chronic conditions could affect the patients’ quality of life [41]. However, many studies have found that a large percentage of health professionals lack confidence, feel inadequately prepared or lack training materials to implement SMS in their routine practice [33]. In Australia, SMS has been difficult to implement within general practice due to high patient volume and time constraints within that setting [41].

Most SMS occurs within primary healthcare settings such as General Practice (with care primarily delivered by GPs and nurses) or within Community Health Centres and Community Aged Care Services (with care primarily delivered by nurses, a broad range of allied health professionals, and support workers). For health professionals in these settings, a key focus is support for patients with chronic conditions; however, it is suggested that GPs, for example, might not prioritise SMS skills or be familiar with SMS principles, especially motivational interviewing, despite behaviour change skills being identified in chronic condition management guidelines as core skills for GPs [42].

Working with patients with chronic and complex health conditions requires particular skills and experience, including highly developed communication and person-centered care skills to facilitate engagement and behavior change, and to work with multi-morbidity. This is likely to mean that training provided to these health professionals is done best using formats that maximize the uptake of these skills; ones that involve significant experiential learning, practice, critical reflection and feedback. Labeau, for example, has argued that e-learning might be less appropriate when the goal is for learners to acquire complex emotional skills [9], such as those required when providing patient-centred care to engage patients with complex chronic care needs in behaviour change to enhance self-management capacity. Recognising this concern, others have proposed the power of storytelling as a teaching and learning strategy within e-learning instructional design to engage learners emotionally and promote deep rather than surface learning [17, 43].

Although the evidence for the effectiveness of e-learning is growing, it is unclear which e-learning instructional designs and formats are best for teaching the depth of skills needed to work with patients with chronic and complex care needs. This literature review was undertaken with this particular lens in mind.

## Methods

An integrative review method was adopted for this literature review, which was conducted at the end of January 2016. This method is broad and is distinguished from other approaches such as systematic reviews, meta-analysis and meta-synthesis because it allows for the researcher to combine data from disparate empirical and theoretical research approaches [44]. It involves four main stages: problem identification; literature search; data evaluation; and data analysis. Applied to this literature review, each of these stages involved the following:

- The problem to be addressed by the literature review

The use of e-learning is now widespread for training and education of health professionals; however, there is a lack of clarity about which e-learning formats and approaches are more or less effective for teaching health professionals chronic conditions self-management support skills and why this might be so.

b) Literature search

A review of the following electronic databases was undertaken: Ovid Medline, Cumulative Index of Nursing and Allied Health Literature (CINAHL), PubMed, Scopus, Proquest, ScienceDirect, Web of Science, Informit and Education Resources Information Centre (ERIC). The search was based on an analysis of possible key text words and index terms used to describe e-learning in the context of SMS and health professionals. Test searches were conducted using identified keywords and index terms. Keyword combinations included: self-management support, self-care support, self-management support skills, self-administration support, self-monitor support, online learning, e-learning, web-based education, blended learning, computer-assisted education, continuing professional development, health professionals, health occupations, allied health personnel, community health workers, occupational therapy, physical therapy, speech-language pathology, medicine, nursing, pharmacy, specialty, podiatry, psychology, medical, nutritionists, physical therapists, and licensed practical nurses.

c) Data evaluation

Inclusion and exclusion criteria are outlined in Table 1 below:

**Table 1:** Inclusion and exclusion criteria

Criteria	Inclusion	Exclusion
1	Published between 2006 and 2016	Published prior to 2006
2	Published in English	Published in language other than English
3	Primary research article	Secondary research article
4	Related to licensed health professionals	Undergraduate students and unlicensed health graduates
5	Related to continuing professional development	Related to patient education
6	Health professionals as the study sample	Patients or undergraduates as the study sample

The second author undertook an initial assessment of the relevance of the papers that were identified by the initial search on the basis of information contained in their title and abstract (*n* = 1505). After removal of duplicates, 52 papers were potentially relevant. The second author then accessed the full text of these papers and reviewed their contents for overall fit with the inclusion criteria. As part of this process, they undertook a further search of the reference sections of these papers to identify other research that may have been missed by the initial search process. Where there was doubt about a paper’s relevance (for example, clarity around what constitutes chronic condition self-management and SMS), they concurred with the other authors to seek clarification on the assessment criteria. A final assessment of relevance was undertaken by the three researchers. They read each potentially relevant research paper and then came together to discuss and reach consensus about papers that fully met the inclusion criteria (*n* = 10), excluding those papers that did not meet these criteria.

d) Data analysis

All papers included in the study were categorised into qualitative, quantitative and mixed methods research, and therefore were separately reviewed using different critical appraisal tools to perform a broad assessment of their quality. To support this critical appraisal, the Joanna Briggs Institute (JBI) QARI critical appraisal checklist for interpretive and critical research [45, 46] was used to appraise qualitative papers; the JBI QARI critical appraisal checklist for experimental studies [45, 46] was used to appraise quantitative studies; and the scoring system developed by Pluye et al. [47] for appraising mixed methods research was used to appraise mixed methods papers. The appraisal process was conducted by the research team who met to discuss and concur on the final appraisal scoring for each paper within each respective checklist. We did not set minimum quality threshold criteria, as our aim was to identify any relevant studies, regardless of their quality. This decision enabled us to show the full range of quality of e-learning studies in the SMS field, even very poor quality studies, to demonstrate the gaps in quality and need for more rigorous research in this area.

A summative content analysis [48, 49] was undertaken by the authors to determine a range of tentative themes from across the included papers; that is, we used a deductive rather than inductive approach. We met to determine tentative theme areas of interest, upfront, and then independently reviewed the studies in order to explore usage, aligned with these areas of interest; known as manifest content analysis. The research team then met to discuss these themes for parsimony, resolving any disagreement through discussion and consensus between at least two of the three authors, and final themes were determined. We then undertook preliminary interpretation of the manifest data; known as latent content analysis, though this was limited by the diversity and small number of identified studies.

## Results

Ten peer reviewed research papers were identified from the review process. Details of the studies, and their aim, sample, methods, major findings and general strengths and limitations are provided in Table 2 below.

**Table 2:** Details of the studies

Author/s location	Sample participants	Delivery methods & E-learning processes	Conceptual framework	Outcome measures	Major findings	Strengths (S) & limitations (L)
Yank et al. (2013) [33] (USA)	(6 groups of 8–14 each) Groups 1–4: 19 internal & 10 family medicine. Groups 5–6: 13 physicians, 7 nurses, 8 allied HPs. Serving patients in community and veteran health centres	Weekly webinars to deliver core intervention using sequential, real-time, interactive, multimedia. Homework assignments between webinars. Groups 1–3 - at least one in-person session. Groups 4–6 - all content delivered by webinar. Access to archived on-line class designed to teach SMS skills. Brief review of prior week’s topic. Introduction to new skills and role modeling of skills by facilitator, participant activities to practice skills (role playing), introduction to the homework assignment (real-world applications of SMS skills between sessions in clinical and online patient class), and wrap up. Week 1: Formation of an achievable action plan Week 2: Problem solving Week 3: Reinterpretation of symptoms and modelling of all prior skills with patients Week 4: Learned for the application in the clinic	Bandura’s theory of self-efficacy	Primary: Changes over time in both beliefs and confidence regarding SMS, measured by retrospective pre/post intervention survey questions (10 point Likert scale) Secondary: Session attendance rates and exploratory focus group qualitative data by semi-structured interview	74% attended 3 or more of the 4 learning sessions; enhancement in the performance of action planning/ positive attitude to SMS/ desire patient involvement and partnership/ desire for other providers to have the training/ reduced burnout	(S): Addressed an unmet need (L): Convenience sample, survey not externally validated, pre/post survey design/response drift, baseline exposure to SMS training not assessed, focus group data analyzed by single researcher
Heartfield et al. (2013) [60] (Australia)	500 practice nurses Serving veterans in primary care	Used evaluation data from a national education and training program; interactive modules offered online provided at no cost to HPs and organization; training session delivered as a sequence of screens presenting visual and auditory information: graphics, content-related additional web links, supporting resources, quiz assessment, free text, true/false responses. Formative learning approach with content, practice and assessment linked by aim and objectives; learners type their responses to learning activities, with automated feedback provided by onscreen text.	Cognitive Behavioural Therapy Motivational Interviewing	Formal and informal qualitative evaluation data: learning needs, relevance of training to practice, clarity of instructions, design and access to resources; open-text comment on what worked well and what needed to be changed to improve the learning experience.	Identified benefits: A new and more patient-centred approach identified and recommended; flexibility in finding time, time saving. Identified challenges: access, navigation, and time.	Not identified (L): Evaluation methods
Welch (2014) [51] (UK)	All registered nurses in any capacity on 3 medical-surgical units and 1 telemetry unit Serving hospital inpatients	Online learning module regarding use of brief motivational Interviewing as a communication style to influence health behaviour change. Estimated 1 h or less for the entire activity. Topics included understanding chronic disease, patient engagement and nurses’ responsibility for promoting health; health behaviour change model, principles of MI, examples of OARS communication skills, scenario about discharge education with and without MI.	Rogers 5 stage decisional process- innovations theory Extended theory of planned behaviour (Côté et al. [80])	Pre/post assessment tool: 6-item questionnaire for MI (Spollen et al. [81]); an attitude survey including 3 items with a 5-point Likert Scale. 2-week data evaluation period.	Positive attitudes toward and statistically significant increase in mean score about effectiveness of online learning modules for MI	(S): Facilitators and role models that might include experienced existing staff in various areas; use of train-the-trainer approach with designated unit staff (educators and first-line managers); (S): Staff completed the module during paid work time: (L): Need more short and long-term outcome measures (L): Project conducted in a single facility
Newton et al. (2011) [42] (Australia)	13 GP supervisors; 13 medical educators; 40 GP Registrars Serving osteoarthritis (OA) patients in primary care	Module: web-interface using the concept of ‘rooms’ (learning material to be streamed into 3 distinct areas) (1) The library room: reading, references, websites, and guidelines to explore knowledge; (2) The consultation room: interactive case studies where GP can engage with patients both online and in an interactive workshop- short interactions about 20 min based on the GP’s preferred learning style (blend learning); and (3) The project room: 3 investigative approaches - patient education, practice quality improvement and learning from patients to increase chronic condition self-management (CCSM) and lifestyle risk modification (LRM) understanding. Detailed manual for GP supervisor; MI: online learning and workshop on motivational interviewing (MI) built around two case studies of 10 min consultations using brief MI techniques. Online quiz for self-knowledge of OA, CCSM and LRM, and immediate feedback.	4 themes identified through the literature review and scoping exercise and encapsulated by the nationally defined Capabilities (Lawn & Battersby, 2009 [34])	(Qualitative and quantitative) Pre/post survey with open and closed questions. MI workshop feedback using 5-point Likert scale questions. 3 qualitative questions: ‘What I learned’, ‘What was a challenge’, and ‘The best part was’.	82.5% of GP registrars considered themselves already well prepared for CCSM and LRM. Supervisors confirmed need to improve CCSM and LRM and lack of skills in this area. Increased confidence in SMS. Immediate feedback; digestible and reasonable size of information; positive learning experiences on MI and highlighted the effects of patient simulation for consultation skills. Technical difficulties.	(S): (L): Low enrolments. Short timeframe of the study (4 weeks). Need for supervision and development of SMS competencies at all stages of health professional development. Low engagement with some aspects of the website.
Bosnic-Anticevich et al. (2014) [53] (Australia)	Pharmacists, GPs and practice nurses and 234 people with asthma Serving patients with asthma in community clinics	Parallel group, repeated measure design. 3 continuing education models (2.5 h workshops or Model 2 online) for HPs to educate patients in correct use of their inhalers and equip the HPs with skills to engage in collaborative relationships with each other. 3 intervention groups and 1 control group; after completing the education module, HPs recruited 10 people with asthma into the study and followed them up for 6 months. The online module modelled the best practice in skill teaching, using a process of instruct-demonstrate-practice, which focuses on the teaching factors that influence learning of motor skills. Scenario-based vignettes showed a HP and patient interaction where all the skills about the inhaler use were explained; HPs observe patients making errors and suggest appropriate action for correction.	Focusing on individual transformation in the clinical context of inhaler technique mastery and maintenance.	Patient asthma outcome and inhaler technique control: patients asked to complete a 6-question asthma control questionnaire. HPs completed: The attitudes toward health care teams scale questionnaire; semi-structured interviews; Inhaler technique checklist developed for this study within 1 week after the module completion.	Protocol paper. Potential challenges identified in the online module: participants could miss out on the interaction and demonstration of correct inhaler technique with fellow participants. Challenge of mirroring the educational content of the models in 3 different learning styles.	(L): Significant financial resources for development of study materials and longitudinal involvement of HPs. Large scale of study could pose challenges for recruitment.
Bowler (2010) [61] (UK)	31 community Matrons Serving patients in community primary care	One hour e-learning CD using cartoon character (STAN) to represent a patient with chronic conditions and how the HP can help patients promote self-care. (STAN case study = Skills, Tools, Advice, Networks offered to the patient) The STAN tool features promotional self-care aids such as tape measures, drinking bottles and pedometers to raise self-awareness of self-care activities. It provides examples of how HPs can support patients in the areas of care planning, goal setting, self-monitoring devices, patient education, home adaptions and peer support networks.	7 core self-care principles (Skills for Care and Skills for Health, 2008)	2 questionnaires used to gather feedback: focusing on the tool’s accessibility and its content.	Participants had little difficulty accessing and going through the online learning module; 45% learnt some new information; mainly a reminder of what was learned in the past.	(S): Involvement of staff in development, piloting and roll out of the tool. (L): Small pilot in one context.
LeRoy et al. (2014) [55] (USA)	Clinicians (professionals unspecified) Serving populations with chronic disease across healthcare	Development of a multimedia library of action-oriented SMS resources and 3 companion videos illuminating SMS skills and concepts, illustrating what SMS is, why it is important and how to provide it in a clinical setting; and illustrating the patient role, building relationships, sharing information, collaborating on agenda setting, goals and action plans, problem-solving and follow-up. An initial environmental scan was conducted to develop the resource library: involved a technical expert panel of 10 clinicians reviewing findings from searched literature; 3 clinicians and 1 clinician-patient pair interviewed; 12 clinicians: short self-administered feedback	An environmental scan.	Expert panel of 10 clinicians and patients participated in a 1-day meeting to review all scan materials. Videos tested by being shown at the Annual AHRQ Conference, USA. 3 clinicians and 1 clinician-patient pair interviewed; 12 clinicians voluntarily viewed videos and completed short self-administered feedback form.	Outcomes of scan: 17% of SMS resources were interactive; 13% were videos (eg. MI, group visits, behavior change); most resources were print materials. Clinicians suggested adding more images about patient interactions and improving interactivity.	(L): Need for translating tools into languages other than English and Spanish; and customizing tools for specific ethnic groups, developing tools beyond action plans, creating materials for non-physician providers and staff.
Wheeler et al. (2013) [79] (Australia)	Pharmacists and pharmacy staff: Serving mental health patients and carers	Multi-step planning and delivery process. Online mental health and education training program for community pharmacy staff using intervention mapping to improve the outcomes for mental health consumers and carers. Techniques include lectures, PowerPoint presentations; question and answer interaction with live audience; resource list, web links, reading material, Previously recorded role plays of staff-patient interactions, discussion, case vignettes, problem-solving tasks. 8 online modules (4 of these for pharmacy staff/8 for pharmacists), each of them taking 30 min to complete followed by a multi-choice assessment taking 5 min to complete; participants must answer all of the questions correctly to pass the test and proceed to the text module, but the test can be repeated as many times as necessary, a certificate is awarded on completion. 6 steps: Needs assessment, program objectives, theory-based methods and practical applications, program planning and development (face-to-face), adoption and implementation plan (online), plan evaluation.	Intervention mapping based on 3 primary activities: Needs assessment (NE), program planning and development (PPD), program evaluation (PE).	Baseline pre-training measures administered to assess knowledge, skills, attitudes and behaviours of pharmacy staff, with a questionnaire to explore reflective learning 6 months post-training. (NE) Interviews with 74 mental health consumers and carers, 15 key stakeholders, and survey with 504 pharmacy staff. (PPD) Face-to-face pilot with 24 pharmacy staff and focus group feedback. (PE) Interviews with 211 consumers and carers, online questionnaire with 282 pharmacists and 222 pharmacy staff.	It allowed the health educators and researchers to approach the education program in a systematic stepwise manner and bring their wide range of theoretical, practical and experiential contributions together to make decisions. Actual outcomes of training not reported in this paper (pending).	Some view intervention mapping as a protocol rather than a guide that is flexible and assists with the decision-making process to meet developers’ needs and circumstances. The process can be cumbersome and time consuming.
Sassen et al. (2014) [52] (Netherlands)	69 HPs (nursing & physiotherapy) Serving cardiovascular patients	Web-based intervention to increase patient intention and risk reduction behaviour toward cardiovascular risk. Several online modules to increase health professionals’ awareness of their thoughts, and learn skills and strategies to support patients’ self-management, such as listing the pros and cons of encouraging patients in the short and long term; supporting behaviour change, feedback system on the progression of the behaviour change.	The Theory of Planned Behaviour	RCT Self-assessed questionnaires. Social-cognitive determinants, intention and behaviour were measured pre-intervention and at 1 year follow-up.	No significant effect detected from the intervention group where the online learning package delivered, no significant differences detected between the two groups.	(L): Low rate of enrollments, didn’t use website intensively due to time and organizational constraints.
Ruiz et al. (2006) [54] (USA)	38 licenced practice nursing students Serving patients with dementia	Dementia computer-based training: aimed to improve knowledge, self-efficacy and attitudes by providing a combination of theory, laboratory, and clinical course work. 7 CD-ROM training modules (20–30 min each): topics included understanding dementia, communication, distress behaviour, loved ones’ activities to daily living, environment, and ethics. Incorporates a variety of content presentation formats to target different learning styles, including text, animations, video, audio, and interactive exercises.	Not identified	Questionnaire to test knowledge and attitudes administered immediately before and after the CD-ROM training. Knowledge measured with 24-item quiz that contained true-false questions; self-efficacy assessed with a 7-item questionnaire (5-point Likert scale). Post-training feedback questionnaire.	Significant improvements in all 3 areas: knowledge, self-efficacy, attitudes. Positive ratings on utility, usability and satisfaction with training modules. Quality of software teaching materials could contribute to development of reusable learning objects that can also be used in blended –learning approaches to improve effectiveness and efficiency of training.	(S): Easy to use; rich multimedia (L): No control or comparison group; generalizability limited by homogeneous sample.

### Critical appraisal

Five of the 10 studies used qualitative methods. Qualitative research presents more subjectivity than quantitative research as qualitative approaches are established on diverse understandings of knowledge. Also, how the researchers interpret the data can influence the intervention results and the generation of study findings. For this reason, critical appraisal should focus on the congruity between study method, data representation and results interpretation. The JBI QARI Critical Appraisal Checklist [46] addressed each of these areas of concern. Table 3 provides the 10 questions that comprise this checklist and an assessment of the five qualitative studies against this checklist.

**Table 3:** The JBI QARI critical appraisal checklist for interpretive and critical research (Pearson et al., [46])

Checklist questions	Study 2 (Heartfield et al. 2013) [60]	Study 3 (Welch, 2014) [51]	Study 4 (Newton et al. 2011) [42]	Study 6 (Bowler 2010) [61]	Study 7 (LeRoy et al. 2014) [55]
1. There is congruity between the stated philosophical perspective and the research methodology	√	√	√	√	√
2. There is congruity between the research methodology and the research question or objectives	X	X	√	X	√
3. There is congruity between the research methodology and the methods used to collect data	Unclear	X	√	√	√
4. There is congruity between the research methodology and the representation and analysis of data	√	√	√	√	√
5. There is congruity between the research methodology and the interpretation of results	Limited	√	√	√	Limited
6. There is a statement locating the researcher culturally and theoretically	X	X	X	X	X
7. The influence of the researcher on the research, and vice-versa, is addressed	X	X	Unclear	X	X
8. Participants and their voices are adequately represented	X	X	Limited	√	Limited
9. The research is ethical according to current criteria or, for recent studies, there is evidence of ethical approval by an appropriate body	X	X	√	Unclear	√
10. Conclusions drawn in the research report do appear to flow from the analysis, or interpretation, of the data	√	√	√	√	√

Three of the 10 studies used quantitative methods. Their appraisal (see Table 4 below) indicates that none of the three studies met all of the appraisal tool criteria. The description of whether the treatment allocation was blinded to participants was absent in all three studies. Table 4 provides the 11 questions that comprise this JBI QARI checklist for quantitative research and an assessment of the three quantitative studies against this checklist.

**Table 4:** The JBI QARI critical appraisal checklist for experimental studies [46]

Checklist questions	Study 8 (Wheeler et al. 2013) [79]	Study 9 (Sassen et al. 2014) [52]	Study 10 (Ruiz et al. 2006) [54]
1. Was the assignment to treatment groups random?	√	√	X
2. Were participants blinded to treatment allocation?	Unclear	Unclear	X
3. Was allocation to treatment groups concealed from the allocator?	Unclear	√	Unclear
4. Were the outcomes of people who withdrew described and included in the analysis?	Unclear	√	X
5. Were those assessing outcomes blind to the treatment allocation?	Unclear	Unclear	X
6. Were the control and treatment groups comparable at entry?	√	√	X
7. Were groups treated identically other than for the named interventions?	√	√	Unclear
8. Were outcomes measured in the same way for all groups?	√	√	Unclear
9. Were outcomes measured in a reliable way?	√	√	√
10. Was there adequate follow-up (>80%)	X	√	X
11. Was appropriate statistical analysis used?	√	√	√

Two of the 10 studies used mixed methods. Table 5 (see below) provides the checklist of 15 questions designed to concomitantly appraise the methodological quality of qualitative, quantitative and mixed methods. The study by Bosnic-Anticevich et al. met all the appraisal criteria [50], while the quantitative experimental part of the study by Yank et al. was presented with insufficient description [33].

**Table 5:** Appraisal checklist for mixed methods research [47]

Type of mixed methods study	Methodological quality criteria	Study 1 (Yank et al. 2013) [33]	Study 5 (Bosnic- Anticevich et al. 2014) [53]
1 Qualitative	• Qualitative objective or question • Appropriate qualitative approach or design or method • Description of the context • Description of participants and justification of sampling • Description of qualitative data collection and analysis • Discussion of researchers’ reflexivity	√	√
2. Quantitative Experimental	• Appropriate sequence generation and/or randomization • Allocation concealment or blinding • Complete outcome data and/or low withdrawal/drop-out	Unclear	√
3. Quantitative Observational	• Appropriate sampling and sample • Justification of measurements (validity and standards) • Control of confounding variables	√	√
4. Mixed Methods	• Justification of the mixed methods design • Combination of qualitative and quantitative data collection-analysis techniques or procedures • Integration of qualitative and quantitative data or results	√	√

### Results of content analysis

Seven main themes were derived from the content analysis. These are outlined in Table 6. Studies involved a diverse range of health professionals, including medical, nursing and allied health professionals (theme 1), matching all those professionals that are important for delivering SMS. Online learning timeframes were of varying length (theme 2). The contents of online learning packages were also varied and covered a broad range of important SMS capabilities [34, 35] including problem-solving, action planning, motivational interviewing, and goal setting (theme 3). Seven of the 10 studies noted a guiding theoretical framework to underpin the rationale for how the training was delivered and its foci. The Theory of Planned Behaviour was noted by two studies [51, 52] and psychology-based theories of behaviour change support appeared to underpin most training (theme 4). Surveys and semi-structured interviews were the main form of evaluation of learning and these focused on broad and more immediate subjective outcomes such as satisfaction, perceived intention to change practice, and perceived change to practice; that is, a comprehensive, multi-lens evaluation of longer-term impact of the e-learning on practice was not evident from the studies. Of note, five studies reported measuring patient outcomes, though the rigor of these measures was unclear [3] (theme 5). Several instructional design features or formats were identified and these reflected the desire to make the e-learning as engaging and interactive as possible [2], and readily linked to real-world practice (theme 6). All 10 studies identified a range of barriers to e-learning that were also applicable to online learning in general (theme 7); though few solutions to these barriers were proffered.

**Table 6:** Themes and subthemes identified from the findings of the studies

Themes	Subthemes	No. of studies	Empirical sources
1. Participants and professions	Nurses	6	[33, 51–54, 60]
	Physicians/General Practitioners	2	[33, 60]
	Allied health professionals	3	[33, 42, 52]
	Primary care residents	1	[33]
	GP registrars	2	[42, 51]
	Clinicians- not specified	2	[51, 55]
2. Online learning time length	Multiple sessions of 20 min or less	1	[42]
	8 × 30 min	1	[79]
	7 × 20–30 min	1	[54]
	Multiple short integrated consultations	1	[52]
	3 short videos	1	[55]
	Not stated	1	[51]
	4 weekly × 60 min	1	[33]
	1 h+	2	[53, 61]
	4 × 6 h	1	[60]
3. Online learning package content	Problem-solving	4	[42, 51, 55, 79]
	Competencies of self-management	6	[42, 51, 52, 55, 60, 61]
	Motivational interviewing	3	[42, 51, 55]
	Community counseling	2	[33]
	Lifestyle Modification	1	[42]
	Action Planning	4	[33, 42, 52, 61]
	Reinterpretation of symptoms	2	[33, 79]
	Asthma inhaler technique correction	1	[53]
	Goal setting	4	[33, 42, 55, 61]
	Dementia education	1	[54]
4. Guiding theoretical framework	Bandura’s theory	1	[33]
	Flinders CCSM Program	2	[42, 60]
	The theory of planned behaviour	2	[51, 52]
	Intervention mapping	1	[79]
	Rogers [82] 5-stage decisional process- innovations theory	1	[51]
5. Outcome Measurements	Retrospective pre/post intervention questions	5	[33, 42, 51, 61, 79]
	Semi-structured interview	5	[42, 51, 53, 55, 79]
	Patient outcome	5	[33, 51, 53, 55, 60]
6. Identified online learning features or format (instructional design)	Webinar	1	[33]
	Resource Library	2	[33, 42]
	Online Video	5	[53, 60, 79]
	Scenario-based Learning	3	[51, 60, 79]
	Intervention Mapping Framework	1	[79]
	Interactive Modules	3	[52, 54, 60]
	Visual and Auditory information	2	[60, 61]
	Web links	3	[52, 60, 79]
7. Identified online learning barriers	Computer Literacy Skills	1	[60]
	Access	9	[4, 51–55, 60, 61, 79]
	Time	10	[33, 42, 51–55, 60, 61, 79]
	Limited Space	2	[53, 60]
	Personal skills of information selection	3	[51, 52, 79]
	Negative Emotions	1	[33]
	Navigation	1	[5]

## Discussion

Returning to the aim of this integrative review, the results show that the evidence remains unclear about which e-learning styles and formats are best for teaching the depth and range of skills needed for health professionals to work with patients with chronic and complex care needs to support their self-management of their chronic conditions. Apparent from these studies is that context is important. Although e-learning has many benefits generally that are also applicable to health professionals involved in the delivery of SMS, completely online delivery of learning within this context poses some problems, with limitations on the scope and types of SMS capabilities that can be taught exclusively and effectively using this format. In particular, practice and application opportunities, and time for reflection built into the learning process were identified as important components for learning about SMS.

Blended learning was valued; that is, combining online learning components with face-to-face learning and practice within real-world practice contexts that also provided opportunities within the safety of the seminar or workshop learning space for reflection with peers, exchange of ideas, and feedback from patients, where available. The participants in Welch et al.’s evaluation of online motivational interviewing training – a commonly addressed curriculum topic in SMS training - stressed the importance of subsequent practice and reflection in the work context following the learning activity, as critical to reinforce skills [51]. The authors argued that there is insufficient evidence to identify an optimal approach to motivational interviewing training, though a classroom format followed by simulated or real-life scenarios is commonly expressed in the literature. Newton et al... found that a hybrid approach was preferred, using a combination of online and face-to-face learning for motivational interviewing – the former providing conceptual knowledge of the required skills and the latter providing the opportunity to practice these skills [42]. Bosnic-Anticevich et al’s study comparing three models of delivery of training to staff in General Practice Networks in Australia to support patient asthma medication use (face-to-face standard, online, and face-to-face interprofessional education) found that technique demonstration was challenging for participants in the online format and therefore did a follow-up interaction with learners to address this [53]. Ruiz et al. supported the use of computer-based training in conjunction with face-to-face training to improve effectiveness and efficiency of education for the long-term care workforce [54]. These results demonstrated that, for some chronic conditions, some aspects of learning still require face-to-face delivery to demonstrate and practice SMS skills.

These aspects of learning about SMS were perceived as difficult to create within a completely online learning space. High levels of creativity in the use of discussion boards, audio-visual resources, and other online teaching tools would be needed to replicate the depth of learning suggested by the evaluation of these studies. Reasons for this may go to the very heart of the definitions of self-management by the patient and SMS by health professionals because person-centred care is the fundamental underlying philosophical stance involved here, and knowing whether we are working in a person-centred way can only ultimately come from the feedback we receive from patients. This is arguably the most powerful learning tool within the SMS learning space. For example, LeRoy et al.’s environmental scan of SMS learning resources found that 17% were interactive web-based tools and 13% were videos (eg. of motivational interviewing); however, most were print materials, in English [55]. They developed three brief videos to illustrate ‘what, why and how’ to do SMS. When they tested these with health professionals, they found that more videos of patient interactions were needed because they were deemed by participants as the most compelling methods to foster their learning.

Sassen et al. appear to have understood this need for practical, ‘real world’ examples of worker/patient interactions and tried to overcome it because their study of online learning to support decision-making and self-management attempted to actively bring both health professionals (nurses and physiotherapists) and their patients into the online learning space. They did this through a series of four short modules completed together, aiming to integrate the clinical care provision space and the learning space [52]. Their results were quite mixed and ultimately showed poor uptake of this intervention, with less than half of intervention participants using the online modules, and no differences in behaviours and intentions found between them and the control group who did not undertake the online learning. The way the information was presented was proposed as the reason for this study’s low participation and high dropout. The quality of web-based materials and their ease of use across varied learner populations, with differing technological experience and access, present significant challenges in creating SMS resources.

Interactivity also appeared to be a particular importance to learning for SMS, and has been noted for e-learning and face-to-face learning, generally [2, 50]. For example, McLeod et al’s study of web-based Interprofessional Psychosocial Oncology continuing education, found that a web-based platform with real-time seminars, discussion boards and multiple audio visual resources that privilege first person illness narratives were important elements in expanding knowledge and changing attitudes about interprofessional practice and person-centred care [56]. Ladhani et al’s study of online modules with use of role-play found that it encouraged distributed participation [57]. A Canadian study of online inter-professional education modules [58] concluded that there needed to be dedicated time and space for e-learning in the work environment, and that e-learning needed to be integrated with existing professional development activities and opportunities for learners to practice the skills learned online. In their discussion paper on best practices for learning with technology, Pilcher & Bradley stressed the importance of interactivity during the learning process, problem-solving, application to prior knowledge and experience, reflection, and also multiple senses stimulation as important for the development of higher order thinking [59]. They argued that, “Interactivity is the key. Participants must interact with the content to retain knowledge and apply it in the work setting” (p.133; see also [2]). They noted the emerging science of ‘brain-based learning’, that is, design that recognizes difference between working memory and long-term memory storage and the types of learning activity design used.

A systematic review of 72 studies of e-learning in medical education by Lewis et al. [19] made a number of recommendations to improve the rigor of e-learning. These included: well-defined goals and objectives determined by a clear understanding and assessment of learners’ needs; careful consideration of situational factors such as learner characteristics, resources and infrastructure, sustainability, and alignment with other educational programs; rigorous evaluation of effectiveness built into the design of the e-learning activity from its inception; it should be based on sound pedagogy to enhance and ensure critical thinking and reflection skills, use cutting edge technologies, and accommodate different learning styles, needs and preferences [19].

The findings of this present review were consistent with Lewis et al’s previous review [19] in that they confirmed a number of known practical benefits of e-learning which were again linked to the profession, context and purpose of the learning, and which SMS capabilities they were designed to teach. For example, Newton et al’s study with GP registrars relied on self-directed learning, with e-learning activities designed to enable short learning sessions of 20 min or less, tailored to the preferences of this professional group [42]. Alternatively, Heartfield et al. designed online modules targeted primarily at GPs which have been predominantly accessed by nurses working within the General Practice setting [60]. The modules are each up to 6 h duration with the functionality to save progress, providing learners with the opportunity to repeatedly re-enter the online site to complete the modules, i.e. stop and start learning. Allowing learners to “go back and forth, double check and pick up where [they] left off” was highly rated [60] (p.289). The flexibility afforded by e-learning was valued in the General Practice context where time constraints were of particular concern and it is recognized that learners in that setting would usually complete the modules outside of working hours. Participants also valued the flexible, self-directed ability of e-learning, and importance of authentic scenarios. Bowler’s study involving community matron nurses undertaking online training in SMS found that this format was useful to reinforce existing skills rather than learn new skills [61]. Ruiz et al.’s study of e-learning for nursing home/aged care staff in Alzheimer’s disease care noted the lack of qualified trainers and time and financial pressures associated with releasing staff for training meant that e-learning offered potential solutions [54]. Of the nursing participants, 92% were experienced computer users. The seven modules were relatively short (20–30 min each), with a clear format that included an introduction, identification of issues and potential solutions, with a practical exercise to finish (a true/false quiz). Although they did not measure impact (only knowledge, self-efficacy, attitudes/satisfaction and intention to use in practice), they found significant improvement in self-efficacy and attitudes towards care, and satisfaction with the e-learning format; it seemed to suit this workforce and context well (busy people on the run). A rich multi-media and high level of interactivity was also important.

The profession, the context and the target of learning have been shown to be important in existing studies of health professionals and e-learning. For example, a Dutch study with vascular surgeons [62] found that they valued the interactivity and videos of techniques, in particular, and expressed a strong interest in blended learning workshops involving preparatory modules followed by a face-to-face workshop practical session for teaching and practicing techniques. A US study [63] of online mandated modules for medical staff to comply with facilities’ requirements is an example of the type of learning that involves compliance and monitoring sign-off of completion for bureaucratic purposes that is suited to online format (see also [64, 65]). A study conducted in developing countries for 98 nurses learning about newborn care [66] found that online combined with hands-on skill enhancement was acceptable and useful and significantly increased their knowledge and skills. Whereas, a UK study of online training in psychological treatments with 183 therapists found that this professional group had little interest in connecting with others online; they were very individual learners who wanted detailed information of how to implement treatments and preferred a ‘human’ host, not an avatar [67]. Alternatively, a Canadian study on adapting online learning for Aboriginal Public Health Workers [68] found that employer support was important for recruitment and retention, as well as use of office equipment and work time, and supervisors showing genuine interest and flexibility. A UK study of 60 healthcare professionals undertaking online learning about patient safety [69] found that the sensitivity of the topic and culture of organizations was a highly debated topic in the discussion boards. Each of these examples points to the importance of authenticity of learning materials to the context in which learning is applied to practice [70, 71].

Other considerations from the broader literature on e-learning are likely helpful to consider for further research in this area. For example, Shaw et al... have proposed a Knowledge-Process-Practice model to guide the educational design of online postgraduate medical education and stressed the importance of applying underlying educational theory to that design, to ensure behaviourist (stimulus and response learning) and cognitivist (perception and the mental transfer of meaning) requirements are met within the content of e-learning materials [72]. Wheeler et al’s study with community pharmacy staff and mental health consumers and carers offers a comprehensive series of intervention mapping steps for development and evaluation that show promise for the development of SMS e-learning [52]. Shaikh et al. [11] and Smith [71] provide useful discussion of the types of learning styles to consider when developing e-learning, drawing on theory developed by Kolb. Smith’s study with 217 nurses found that all four styles (accommodators, assimilators, convergers and divergers) were represented across their sample, though a larger proportion were accommodators; that is, more likely to, “desire hands-on experiences, carry out plans and tasks and using an intuitive trial-and-error approach to problem solving” [73] (p.49).

Lister provides useful tips for effective design of online modules [74]. Although these ideas were not applied specifically to health professions, a number of core qualities of value to health professionals are apparent. These qualities include: ensuring variety of formats to offer choice and engage people with different learning styles; authenticity and reflection to promote coherence and meaningfulness and sustain interest in the learning activity; and built-in learner self-assessment activities to enable immediate feedback, review, higher order thinking and promote successful learning. Likewise, Twigg’s work on blended learning is also worth considering here [75] (see also [76]). Twigg outlined 4 models for structuring e-learning and face-to-face learning options: the Supplemental Model maintains traditional course structures supplemented by e-learning resources and activities; the Replacement Model substitutes some face-to-face contacts with e-learning activities and communication; the Emporium Model replaces formal lectures with e-learning resources to foster enquiry-based learning strategies; and the Buffet Model allows learners to graze through a range of learning environments and activities.

A final consideration is worth noting. Wheeler et al. argued that rigor in determining the content upfront with end-users is important for SMS education [52]. They chose experiential learning techniques with consumer and carer narratives to challenge pharmacists’ held beliefs and attitudes; that is, they, “employed emotional and affective pathways to understanding rather than solely utilizing traditional didactic techniques” [52] (p.262). This approach prompts the question of whether SMS is an area that requires this form of learning to challenge cultural attitudes about health professional expertise and promote deeper learning [17, 43]. Given a central tenet of chronic condition self-management is partnership and that patients and health professionals have expertise to bring to the interaction, such an approach is likely to be warranted. The broad literature on the benefits of consumer involvement in healthcare research, education and practice is well established and would therefore strongly support this recommendation [77, 78].

## Conclusion

The development of e-learning options for the delivery of SMS continuing education to health professionals is a relatively new and growing area, as shown by the limited number of studies identified by this review. There is still much to understand about how best to deliver e-learning in this space. This paper has identified a number of areas that warrant further investigation and suggested that the context of SMS for patients with chronic condition matters to how learning is delivered, to ensure partnership and person-centred care. Further creative approaches and their rigorous evaluation are needed to deliver completely online learning in this space. For now, few e-learning approaches appear to overcome the high levels of interactivity, reflection, practice and application to practice that are needed to learn how to deliver effective SMS to these patients. Blended learning, combining online and face-to-face components, is suggested as the best way forward to overcome these needs.

## Abbreviations

JBI: Joanna Briggs Institute
SMS: Self-management support
